# A randomized controlled trial of endodontic treatment using ultrasonic irrigation and laser activated irrigation to evaluate healing in chronic apical periodontitis

**DOI:** 10.4317/jced.56368

**Published:** 2020-09-01

**Authors:** Akansha Verma, Rakesh-Kumar Yadav, Aseem-Prakash Tikku, Anil Chandra, Promila Verma, Ramesh Bharti, Vijay-Kumar Shakya

**Affiliations:** 1 Professor, Department of Conservative Dentistry and Endodontics, King George’s Medical University, Lucknow, U.P., India

## Abstract

**Background:**

Aim of this trial was to evaluate the combined clinical and radiographic success rate of endodontic treatment using passive ultrasonic irrigation (PUI) and laser activated irrigation (LAI) as compared to conventional syringe irrigation.

**Material and Methods:**

Permanent incisors and single rooted premolars were assessed for eligibility and 69 patients were randomly divided into three treatment groups (n=23) by allocation concealment method and irrigation was performed in accordance with the allocated group. Teeth were evaluated clinically and radiographically with CBCT after 6 months and 12 months of the treatment.

**Results:**

A significant difference was observed in the radiographic healing rates among three groups (χ2=12.29, *p*=0.05). On comparing the final outcome among the three groups (n=19), it was found that 2 (10.5%) cases of group I(Conventional Syringe irrigation), 7 (36.8%) cases of group II (Passive ultrasonic irrigation) and 8 (42.1%) cases of group III(Laser activated irrigation) were healed while under healing category 13 (68.4%) cases of group I, 12 (63.2%) cases of group II and 11 (57.9%) of group III were observed whereas 4 (21.1%) cases were categorised as diseased in group I only.

**Conclusions:**

LAI and PUI can increase the predictability of the endodontic treatment success in cases of chronic apical periodontitis.

** Key words:**Cone-beam computed tomography, CBCT-PAI, Irrigation, LASER, Radiographic healing, Root canal treatment, Ultrasonic.

## Introduction

Apical periodontitis is the most common sequel of endodontic infection. It has been established that the chances of complete healing in teeth with apical periodontitis are 10-15% lower than in teeth without apical periodontitis (1,2). The goal of a root canal treatment is to prevent or to heal apical periodontitis; hence complete debridement of the microbes residing inside the root canal is mandatory to achieve this goal (3). Complex anatomy of the root canal system often results in untouched areas inside the root canal system during chemo-mechanical debridement of the root canal, resulting in failure of the endodontic treatment. In oval canals only 40% area of the apical root canal wall comes in contact with the rotary file (4).

Irrigation plays a pivotal role in the success of endodontic treatment. There are certain requirements that an irrigating solution should possess but unfortunately, none of the currently available irrigating solutions fulfil all the ideal requirements. Hence, for complete disinfection of the root canal, a combination of two or more irrigants in a specific sequence can be used. An ideal irrigation protocol should be efficient in chemical disinfection as well as physical disinfection (detachment of biofilm and planktonic bacteria by exerting shear stresses) on the root canal wall (5).

The efficiency of the irrigating solution largely depends upon the delivery of the irrigant in the root canal and its agitation by irrigant activation systems such as lasers and sonically or ultrasonically vibrating instruments (6,7). During PUI, acoustic energy is transmitted through an oscillating file or smooth wire to the irrigant present inside the canal, and this energy, in the form of ultrasonic waves, induces cavitation and streaming of the irrigant which enhances its potential to contact a greater surface area of the canal wall, resulting in more efficient removal of debris and microorganisms (8,9). As far as Laser activated irrigation (LAI) is concerned, it has been statistically found to be more effective in disinfecting the root canals as it helps better penetration of irrigant into the dentinal tubules (10).

Large number of *in vitro* studies have emphasised on the importance of irrigant activation as compared to few *in vivo* studies. Thus the aim of this study was to conduct a randomized controlled trial to evaluate the combined clinical and radiographic success rate of endodontic treatment using passive ultrasonic irrigation (PUI) and laser activated irrigation (LAI) as compared to conventional syringe irrigation.

## Material and Methods

-Patient Selection: Prior to the initiation, Institutional ethical clearance was obtained to conduct this clinical trial (1225/28/12/2016). The trial was also submitted to the Clinical Trials Registry India (CTRI), under registration no. CTRI/2018/02/012107.

Sample size was calculated using standard formula, keeping the power at 90% and confidence interval at 95% and including the dropouts, a total of 80 patients were selected for the study who reported to the OPD of Conservative Dentistry and Endodintics, KGMU, Lucknow, India, out which only 69 patients fulfilled the criteria of inclusion and exclusion and were randomly divided into three treatment groups (n=23). For allocation concealment, 69 sequentially numbered, opaque, sealed envelopes (SNOSE) containing group information were prepared and handed over to the patient at the time of treatment.

-Eligibility Criteria:

Inclusion Criteria:

•Patients between 18 to 60 years of age.

•Informed consent was obtained from all individual participants included in the study.

•Permanent incisors and single rooted premolars indicated for root canal treatment.

•Teeth with peri-apical score 3 to 5 according to CBCT-PAI (11).

•Patients with good oral hygiene.

Exclusion Criteria:

•Previously attempted pulpotomy or pulpectomy or root canal treatment.

•Immunocompromised patient (i.e. Diabetes mellitus, AIDS, Hepatitis B, Hepatitis C, Tuberculosis, Cancer patients).

•Pregnant females.

•Tooth with a mobility score >2.

•Tooth with a periodontal pocket.

•Tooth with incompletely formed root apices.

•After access opening if drainage from the canal could not be controlled.

•Tooth with internal or external root resorption.

•Tooth with vertical or horizontal fracture extending below the CEJ.

After obtaining consent, the whole procedure of root canal treatment was performed by a single operator to avoid inter operator bias. The assessment of baseline and follow up parameters were performed by different endodontists, blinded regarding the intervention given in a particular patient. Radiographic Technique

Pre-treatment diagnostic intra-oral peri-apical radiographs were obtained to assess the presence of peri-apical pathology using paralleling technique. Preoperative Cone Beam Computed Tomography was performed to evaluate the size of the lesion in all three dimensions as it served as the baseline scan for comparison during follow up visits. The extent of the lesion was marked by the working tools of CS 3D Imaging software (Kodak Dental Systems, Carestream Health, Rochester, NY, EUA) for mesio-distal width, bucco-palatal depth and diagonal dimensions in millimetres, in the coronal, axial and sagittal sections respectively. The maximum dimension among all three sections was used to grade the lesion according to CBCT-PAI introduced by Estrela *et al.* which is a six point (0-5) scoring system (11). Lesions having CBCT-PAI score 3 to 5 were included in the study.

-Root canal procedure

The procedure for root canal treatment was kept same in all the three groups except for the final irrigation protocol. After administration of local anaesthesia, rubber dam was applied for complete isolation of the tooth that had to be treated. In cases of deep caries and trauma the lost tooth structure was restored with composite resin to reproduce the normal anatomy. A straight-line access cavity was made with a sterile Endo access bur (Dentsply). Any necrotic tissue present inside the root canal was removed by irrigating the root canal with normal saline through a syringe with 28 gauge side vented needle. The cases where weeping canals were present and purulent discharge could not be controlled through repeated attempts, were excluded from the study. After access cavity preparation, patency of the root canal was confirmed with a 10 K file and the working length was determined with the help of a radiograph. Protaper Next file system was used for the biomechanical preparation till size X3. Between each instrument change the root canal was irrigated with 2ml of 3% NaOCl. Once the biomechanical preparation was complete, the root canal was copiously irrigated with normal saline to flush out residual NaOCl from the canal.

In group I activation of NaOCl was not performed whereas in group II the root canal was dried with sterile paper points and again flooded with 2ml of 3% NaOCl which was activated ultrasonically for 20 seconds. Ultrasonic activation of 3% NaOCl was performed four times, resulting in 8ml of NaOCl activated for a total of 80 seconds. Ultrasonic activation was performed with an ultrasonic device (P-Max Newtron; SatelecActeon, Merignac, France) by using a #20 stainless steel parallel-shaped noncutting instrument (IrriSafe; SatelecActeon) 2 mm short to the working length. In Group III dried root canal was flooded with 2ml of 3% NaOCl which was activated by 1.5 W Nd:YAG Laser(FIDELIS (AT), Fontana, Slovenia) at pulsed mode of 15Hz for 5 seconds. The optic fiber was kept 5 mm short of the working length and kept steady at this position during activation. This procedure was repeated four times with a total 20 seconds of activation, with the rest interval of 3 minutes between each activation. Laser activation of 3% NaOCl was performed four times resulting in 8ml of NaOCl activated for total of 20 seconds.

1ml of 17% EDTA was delivered into the canal for 1-2 min. After this the root canal was copiously irrigated with distilled water to remove any residual EDTA from the canal. The root canal was dried with sterile paper points. AH Plus root canal sealer was applied onto the root canal walls with the help of lentulospirals and root canal was obturated with the corresponding size gutta-percha master cone along with accessory gutta-percha points by using cold lateral condensation technique. The access cavity was restored with composite resin on the same appointment. During follow up, patients were examined both clinically (at 7th day, 6 and 12 months) and radio graphically (CBCT at 6 and 12 months) to assess the success of the treatment and to determine the amount of healing occurred.

-Clinical evaluation at follow up:

The treated tooth was evaluated for secondary outcome measures. Patients were evaluated for any sinus/ pus discharge and tenderness on percussion. Postoperative pain was evaluated by using the visual analogue scale (VAS), 7 days after treatment, as well as at each re-evaluation visit.

-Radiographic evaluation of healing with CBCT (Primary outcome measure):

Pre and postoperative CBCT-PAI scores and their differences were recorded for all the groups and statistically evaluated. Considering a reduction in CBCT-PAI score as 1 score reduction = 1 level change and so on, data comprising of CBCT-PAI score was categorised into four categories and statistically analysed to determine how much reduction in the CBCT-PAI score has occurred over the period of 6 months and 12 months.

• Unchanged- no reduction in CBCT-PAI score 

• Mild change- up to 2 score reduction in CBCT-PAI score

• Moderate change – 3-4 score reduction in CBCT-PAI score

• High change – more than 4 score reduction in CBCT-PAI score

-Outcome measurement:

The outcome was evaluated by assessing a combined measure of clinical and radiographic components. On the basis of 12-month re-evaluation visit, teeth were classified as healed, healing, or diseased.

1. Healed: Clinical normalcy accompanied by radiographic CBCT-PAI scores of 1 or 2 or no peri-apical radiolucency.

2. Healing: Clinical normalcy accompanied by reduction in the size of the peri-radicular lesion and a reduction in the CBCT-PAI score.

3. Diseased: Presence of any clinical signs and symptoms accompanied by no change in the CBCT-PAI score or an increase in the size of the peri-radicular lesion or an increase in the CBCT-PAI score.

The final treatment outcome was dichotomous (successful and unsuccessful); teeth classified as healed or healing at 12 months follow up were considered successful, whereas diseased teeth were considered as unsuccessful.

-Statistical analysis: The obtained data was analysed using descriptive statistics and making comparisons among various groups by using Chi-square (χ2) test. Discrete (categorical) data was summarized as proportions and percentages (%).

## Results

No significant difference (*p*= 0.840) in mean ages was observed among the groups hence the three groups were age matched. Pre-operatively, no significant difference was found in proportion of various CBCT-PAI scores among the three groups (χ2= 1.158, *p*= 0.997) signifying that the allocation of patients into various groups was randomized, irrespective of lesion size forming similar baseline for comparison.

 Inter observer reliability of clinical and radio-graphical parameters was analysed by Kappa-Cohen test (k value) and the Kappa statistics were found to be k=0.82 at all periods indicating high reliability of observed outcomes.

Total recall rate was 89% after 12 months. At 1 year follow up total number of healed cases were 10.5%, 36.8% and 42.1% in group I, group II and group III respectively whereas 68.4%, 63.2% and 57.9% cases were found to be under healing category ( Table 1).

**Table 1:**
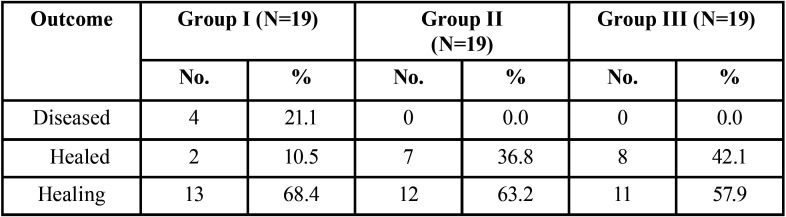
Treatment Outcome of the Three Groups after 12 months.

A significant difference was found in proportion of CBCT-PAI score changes among the three groups at 12 month (χ2=12.29, *p*=0.05) ( Table 2). Group II and III revealed maximum reduction in CBCT-PAI score of peri-apical lesion in 10.5% and 15.8% cases respectively whereas CBCT-PAI score remained unchanged in 21.1% cases of group I ( Table 2).

**Table 2:**
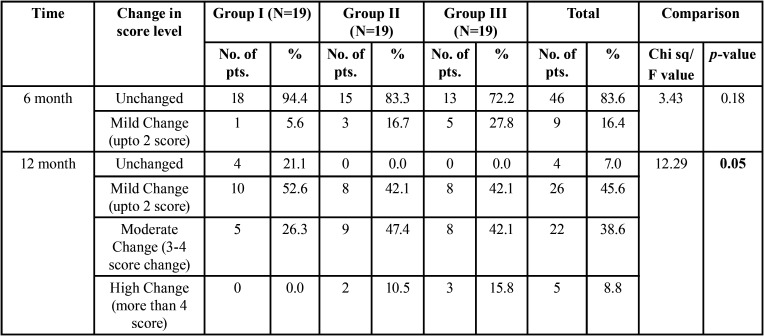
Intergroup comparison of Score Change among three Groups at 6 months and 12 months.

Intergroup analysis regarding reduction in CBCT-PAI score revealed a significant difference in proportion of CBCT-PAI score changes between group I & III at 12 months (χ2=7.91, *p*=0.048) whereas difference in the proportion of CBCT-PAI score changes between group I and II (χ2=7.37, *p*=0.061)and group II and III (χ2=.259, *p*=0.879) were not significant ( Table 3).

**Table 3:**
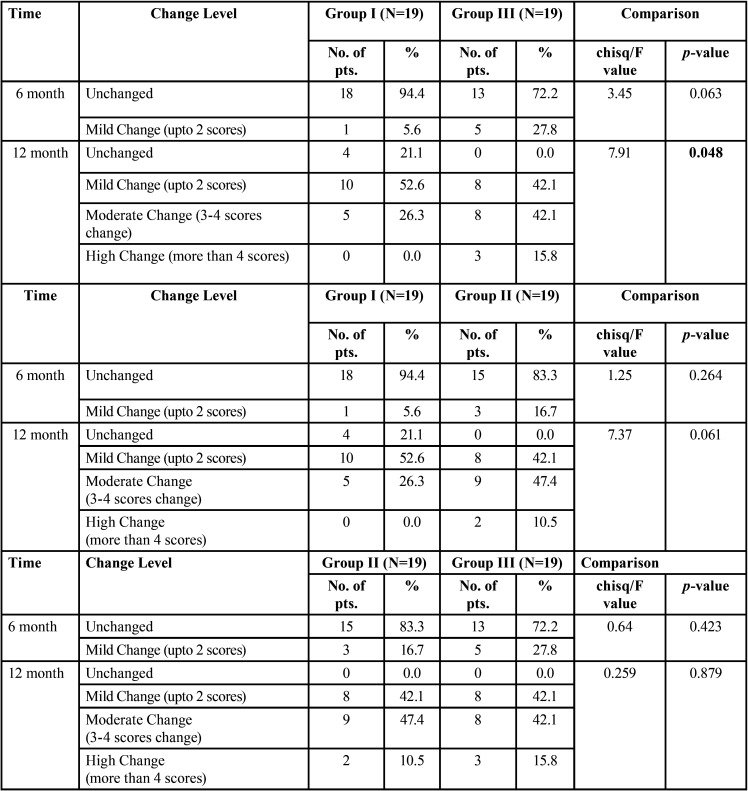
Intergroup comparison of CBCT-PAI Score Change between two groups.

The success rate of the treatment group II and III was equal and 100% while the success rate of group I was 78.9% and found to be significantly inferior (χ2=8.6, *p*=0.014) ( Table 4).

**Table 4:**
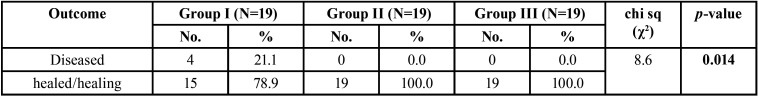
Intergroup comparison of Success rate of the Three Group.

## Discussion

For standardisation of the treatment procedure, only single rooted tooth were included in the study, in single rooted teeth the apical canal is frequently larger than the master file hence the role of irrigation can be stressed upon (12).

IOPA is a 2-D image of a 3-D anatomy, hence it cannot depict the true dimensions of a peri-radicular disease. Moreover 30%-45% of the peri-apical lesions remain undetected in IOPA (13,14). Various recent studies have reported that CBCT imaging is a more reliable tool for measuring and monitoring of the peri-apical lesion (15-19). Hence false positive results related to healing of peri-apical lesions can be avoided with the help of CBCT. Taking this into consideration, CBCT imaging was used to monitor and compare radiographic healing among three treatment groups.

The conventional syringe irrigation group was taken as a positive control group as this technique has got a good success rate when it comes to *in vitro* studies regarding disinfection or *in vivo* studies regarding peri-apical healing (20,21). A total of 4 cases in group I were unsuccessful. Among them 3 cases were considered unsuccessful because the CBCT-PAI score remained unchanged whereas in 1 patient, treatment was held unsuccessful both clinically and radio graphically. A possible cause for the failure in 4 patients can be attributed to incomplete penetration of irrigating solution inside the root canal irregularities, leaving the cause of infection inside the canal. Studies have confirmed the presence of abundant smear in the apical region of root canals even when the irrigation was performed within 1mm of the working length (22,23). although it is possible that incomplete but maximum reduction of bacteria, hermetic root canal filling and a good coronal seal can lead to successful treatment outcome. However the root canal disinfection always remains critical (24).

The overall success rate (healed and healing) of treatment in group II (PUI) and group III (LAI) were 100% as all of the patients revealed some reduction in CBCT-PAI score and none of the patients revealed any untoward clinical finding during 12 month follow up. Ultrasonic activation of the irrigant enhances debridement of the canal chemically (rise in the temperature of the irrigant) as well as physically (acoustic streaming and cavitation of the irrigant) (25-27). However Liang *et al.* demonstrated no significant difference in the reduction of the periapical lesion size when conventional syringe irrigation and PUI were compared (28). Căpută*et al.* in a recent systematic review also confirmed the same (29). Similar results were found in our trial (χ2=7.37, *p*=0.061) ( Table 3) however the overall success rate was higher and significant for PUI group.

As far as LAI is concerned, the antimicrobial effect of the Nd:YAG is based on thermal heating of outer and inner environment of bacteria and its bactericidal effect up-to 1mm inside the dentinal tubules (30). Moreover Nd:YAG laser assisted irrigation is statistically more effective in removing smear layer from the root canal when compared to the syringe irrigation and passive ultrasonic irrigation (31). Masilionyte and Gutknecht in a retrospective study suggested that laser-assisted endodontic protocol is a reliable alternative to conventional treatment, allowing a decrease of chemical irrigation solutions, intracanal medication, and systemic antibiotic use and initiating faster healing of periapical lesion (32).

**Figure 1:**
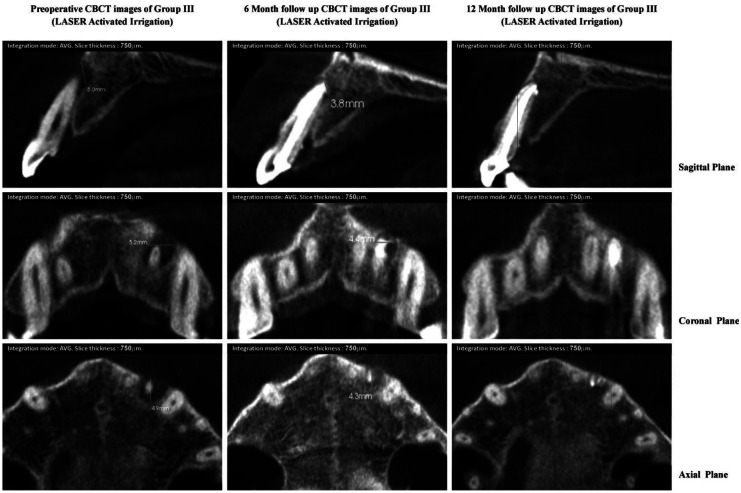
CBCT images of group I (conventional syringe irrigation) showing pre-operative and post-operative dimension of periapical lesion in all three planes.

**Figure 2:**
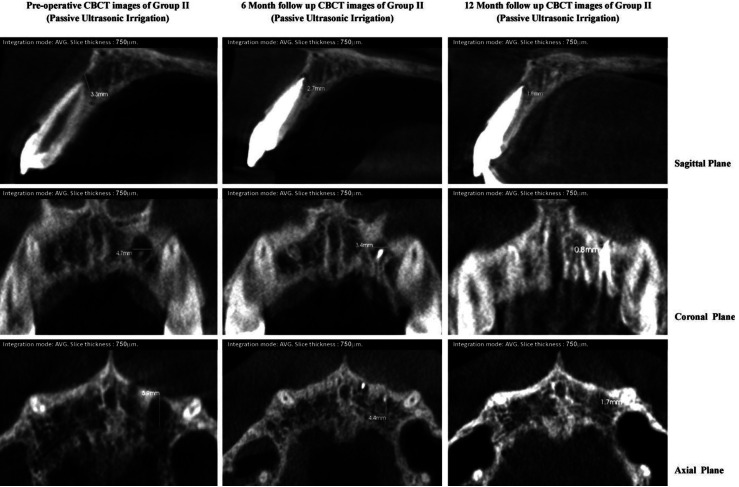
CBCT images of group II (passive ultrasonic irrigation) showing pre-operative and post-operative dimension of periapical lesion in all three planes.

**Figure 3:**
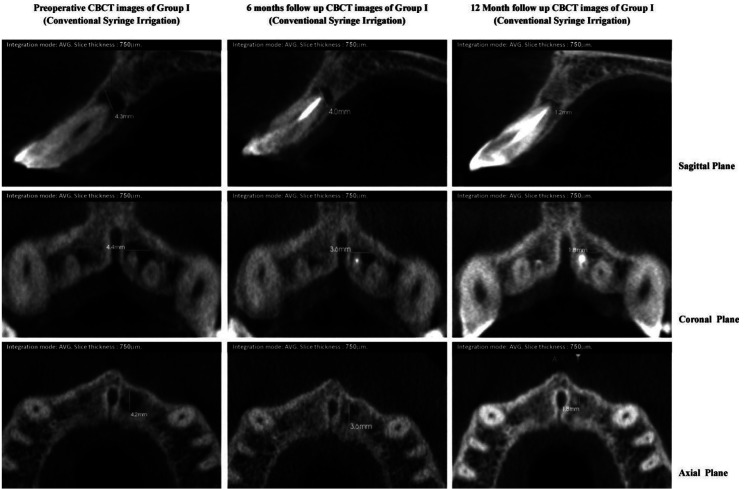
CBCT images of group IIII (Laser assisted irrigation) showing pre-operative and post-operative dimension of periapical lesion in all three planes.

Faster healing was observed in lesions of group II and III, having similar or higher preoperative scores. Group II and III revealed maximum reduction in CBCT-PAI score of peri-apical lesion in 10.5% and 15.8% cases respectively whereas none of the cases in group I revealed high change, signifying faster radiographic healing in the experimental groups. This can be well illustrated by Table II where healing of the peri-apical lesions of LAI group III (Fig. 1) and PUI group II (Fig. 2) occurred at a significantly higher rates than group I (Fig. 3)

A possible cause of the final outcome of 100% success rate in group II and III can be attributed to better disinfection of the root canal by Passive ultrasonic irrigation (PUI) and LASER activated irrigation (LAI).

Periapical pathologies which got completely healed were less in group I i.e. 10.5% whereas in group II and III completely healed cases were more i.e. 36.8% and 42.1% respectively. We did not follow the strict criteria (complete absence of the radiolucency) for the evaluation of success of the treatment (33). The reason was short duration of follow up as the lesions with preoperative CBCT-PAI score of 5 or 5D may take longer duration to heal completely as compared to the lesions of lesser dimensions. Hence final success outcome was based on cumulative assessment of completely healed lesions as well as lesions which have undergone some reduction in the CBCT-PAI score. Moreover evaluation of cases for one year period is supported in literature by various studies. Ørstavikfound complete healing of preoperative chronic apical periodontitis may take 4 years, while signs of initiated healing were visible in at least 89% of cases after 1 year (34).

Use of CBCT as a main assessment tool as more accurate lesion size and their differences can be easily detected with CBCT eliminating the chances of false positive results. High radiation exposure is the major concern related to the use of CBCT, making it to be a possible limitation for the study. However the cumulative effective dose received due to exposure of three CBCT scans was much lower than the dose limit per year (35).

The present study has been designed to overcome potential bias and confounding variables as much as possible. Within the limitation of the present study use of PUI and LAI can be considered as a viable modality in single visit endodontic treatment especially while dealing with cases of chronic peri-apical lesions. Therefore present study might provide the basis for further research in a larger sample size with long term follow up so that the role of irrigant activation can be stressed upon.
